# Supplementary material for: Acute, Low-Dose Neutron Exposures Adversely Impact Central Nervous System Function
Source: Int J Mol Sci. 2021 Aug 21;22(16):9020. doi: 10.3390/ijms22169020 (PMC8396607; doi:10.3390/ijms22169020)
Supplement: Supplementary file 1 [file ijms-22-09020-s001.zip › ijms-1324112-supplementary.pdf]

Supplementary Table S1. Action potential characteristics of CA1 pyramidal neurons are not altered by acute neutron irradiation. Action potential (AP) properties in whole cell current clamp recordings of CA1 pyramidal neurons from the superficial layer of the dorsal hippocampus were measured 3-5 months after acute exposure to 18 cGy neutron irradiation.

Parameter	Mean [95% CI]		Mean Difference [95% CI]	Cohen's d [95% CI]	MLM z-Value	MLM p-Value
	0 cGy	18 cGy				
AP height (mV)	88.9 [81.4, 96.3]	84.1 [76.9, 91.2]	-4.80 [-13.26, 5.01]	-0.36 [-1.14, 0.41]	0.81	0.417
AP width (ms)	0.83 [0.74, 0.92]	0.89 [0.74, 1.04]	0.06 [-0.09, 0.22]	0.28 [-0.49, 1.07]	0.77	0.444
Afterhyperpolarization (mV)	-15.4 [-18.7, -12.1]	-13.4 [-17.8, -9.1]	1.98 [-3.04, 6.54]	0.29 [-0.49, 1.10]	0.75	0.453

Supplementary Table S2. Characteristics of synaptic inputs to CA1 pyramidal neurons are unchanged following acute neutron irradiation. Spontaneous excitatory postsynaptic current (sEPSC) and inhibitory postsynaptic current (sIPSC) properties in whole cell voltage clamp recordings of CA1 pyramidal neurons from the superficial layer of the dorsal hippocampus were measured 3-5 months after acute exposure to 18 cGy neutron irradiation.

Parameter	Mean [95% CI]		Mean Difference [95% CI]	Cohen's d [95% CI]	MLM z-Value	MLM p-Value
	0 cGy	18 cGy				
sEPSC rise time (ms)	2.40 [2.25, 2.55]	2.40 [2.27, 2.53]	0.00 [-0.19, 0.16]	0.00 [-0.76, 0.76]	0.00	0.997
sEPSC charge transfer (pC)	-98.1 [-111.2, -85.0]	-83.3 [-102.3, -64.2]	14.8 [-9.2, 33.2]	0.52 [-0.40, 1.38]	1.47	0.142
sEPSC decay tau (ms)	4.71 [4.00, 5.41]	5.68 [3.82, 7.53]	0.97 [-0.30, 3.57]	0.40 [-0.37, 1.00]	1.04	0.297
sIPSC rise time (ms)	3.28 [3.02, 3.53]	3.39 [3.11, 3.67]	0.11 [-0.23, 0.44]	0.24 [-0.60, 1.02]	0.57	0.570
sIPSC charge transfer (pC)	484.7 [286.6, 682.7]	356.5 [292.0, 421.1]	-128.1 [-385.2, 8.1]	-0.49 [-1.00, 0.30]	0.91	0.364
sIPSC decay tau (ms)	7.59 [6.88, 8.31]	7.10 [6.45, 7.76]	-0.49 [-1.31, 0.36]	-0.42 [-1.14, 0.40]	1.08	0.281
